# Contribution of Soft Substrates to Malignancy and Tumor Suppression during Colon Cancer Cell Division

**DOI:** 10.1371/journal.pone.0078468

**Published:** 2013-10-22

**Authors:** Morgane Rabineau, Leyla Kocgozlu, Denis Dujardin, Bernard Senger, Youssef Haikel, Jean-Claude Voegel, Jean-Noel Freund, Pierre Schaaf, Philippe Lavalle, Dominique Vautier

**Affiliations:** 1 Inserm UMR 1121, Strasbourg, France; 2 Université de Strasbourg, Faculté de Chirurgie Dentaire, Strasbourg, France; 3 CNRS, UMR 7213, Illkirch, France; 4 CNRS, UPR 22, Institut Charles Sadron, Strasbourg, France; 5 Inserm UMR S1113, Université de Strasbourg, Strasbourg, France; 6 Université de Strasbourg, Faculté de Médecine, Strasbourg, France; 7 Fédération de Médecine Translationnelle, Strasbourg, France; Faculty of Medicine University of Leipzig, Germany

## Abstract

In colon cancer, a highly aggressive disease, progression through the malignant sequence is accompanied by increasingly numerous chromosomal rearrangements. To colonize target organs, invasive cells cross several tissues of various elastic moduli. Whether soft tissue increases malignancy or in contrast limits invasive colon cell spreading remains an open question. Using polyelectrolyte multilayer films mimicking microenvironments of various elastic moduli, we revealed that human SW480 colon cancer cells displayed increasing frequency in chromosomal segregation abnormalities when cultured on substrates with decreasing stiffness. Our results show that, although decreasing stiffness correlates with increased cell lethality, a significant proportion of SW480 cancer cells did escape from the very soft substrates, even when bearing abnormal chromosome segregation, achieve mitosis and undergo a new cycle of replication in contrast to human colonic HCoEpiC cells which died on soft substrates. This observation opens the possibility that the ability of cancer cells to overcome defects in chromosome segregation on very soft substrates could contribute to increasing chromosomal rearrangements and tumor cell aggressiveness.

## Introduction

 Over the last 10 years, it has become evident that cell behaviour not only depends on chemical cues but that mechanical properties of cellular environment play an as important role. This was spectacularly demonstrated by the landmark experiments of Discher’s group who showed that mesenchymal stem cells can either differentiate into osteoblasts, fibroblasts or neurons depending upon the Young modulus of the adhesion substrate [1]. It is also well accepted that different cell types need substrates of different Young moduli to properly adhere and proliferate. Osteoblasts require Young moduli in the range of MPa to adhere whereas fibroblasts adhere on softer substrates whose moduli of about 10 kPa [2] and neurons grow on extremely soft substrates of about 1 kPa [1]. These distinctive values are in accordance to the Young moduli that characterize the tissues surrounding these different cell types. These results are of paramount importance for example in tissue engineering to design scaffolds allowing an appropriate growth of cells or in implant integration. Yet adhesion is not the only aspect that characterizes the cell behaviour: cell division is also a crucial aspect for cell fate. Our group started recently to examine the influence of the mechanical properties of the substrate on cell division [3]. These data highlighted that the mechanical properties of the substrate play a critical role in chromosome segregation during mitosis of epithelial cells. Indeed, we observed a progressive increase in chromosomal segregation abnormalities with decreasing substrate stiffness in non-cancerous rat kangaroo kidney cells PtK2 [3]. Moreover, soft substrates (below 50 kPa) were described as a physical microenvironment barrier almost completely inhibiting the PtK2 cells [3].

**Figure 1:**
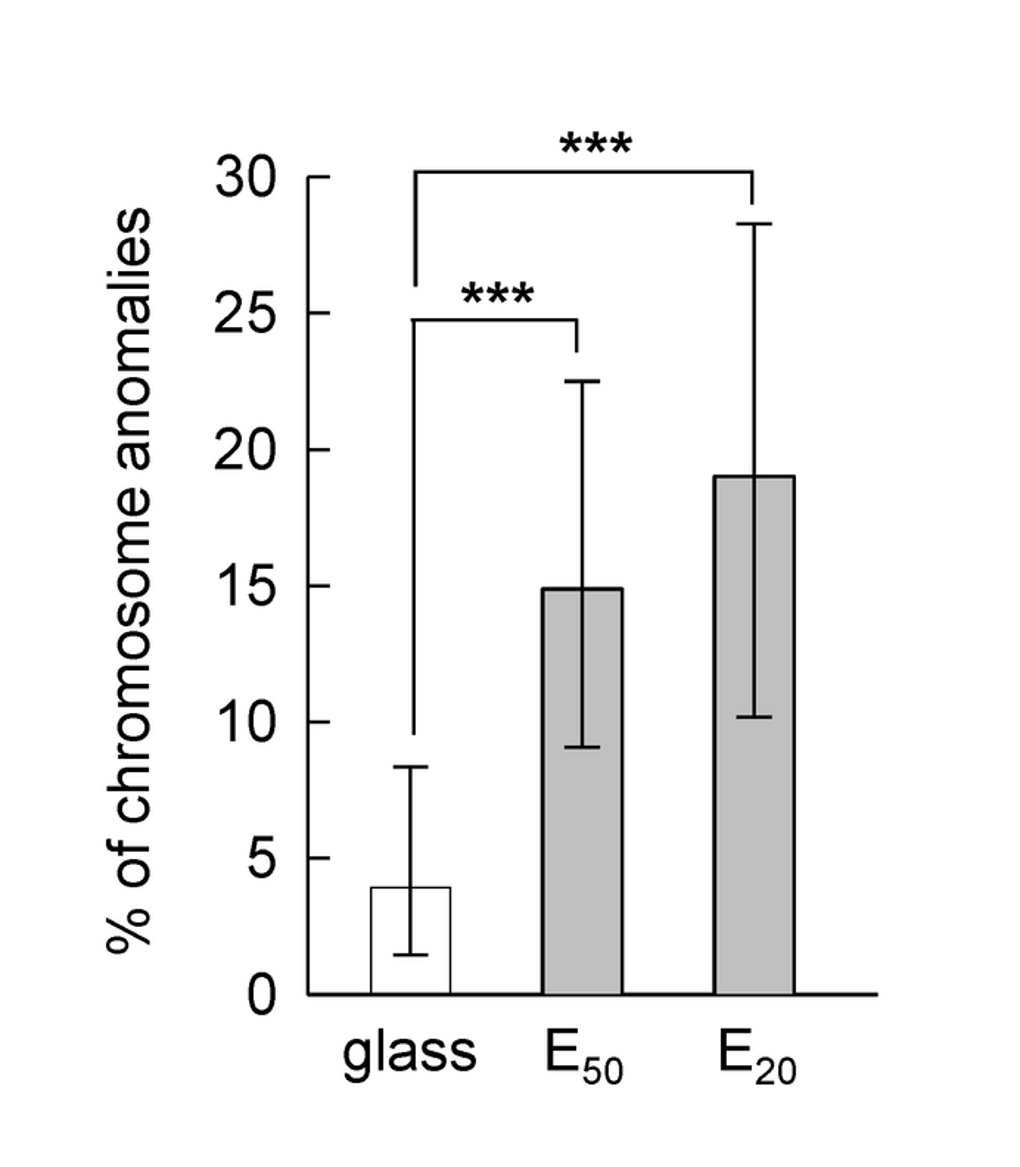
Percentage of SW480 cells 30 min-2h post-synchronization from fixed cells presenting abnormal chromosome morphologies on glass, E_50_ and E_20_, determined on 2 pooled independent experiments. Fisher Exact Test shows the proportion on E_50_ (18 cells with chromosome anomalies on 121 cells analyzed) is significantly different than that on glass (6/153, *p* < 0.003), and the proportion on E_20_ (14/78) is significantly different than that on glass (*p* < 0.001). The errors bars represent 95% confidence intervals.

Over the last years, it has been established that tissue stiffness influences tumor progression and can promote the malignant behaviour [4-6]. By introducing cancer cells into 3-dimensional fibrin matrices, Liu et al. showed that soft matrices of Young modulus about 100 Pa promoted the growth of round colonies with increasing aggressiveness when xenografted in immunodeficient mice [7]. Very recently, Tang et al. revealed the attenuation of cell mechanosensitivity of tumor cells when cultured on soft substrates [8]. In colon cancer, a highly aggressive disease, progression through the malignant sequence is accompanied by increasing chromosomal rearrangements [9-12]. To colonize target organs, invasive cells cross several tissues of various elastic moduli (as example, 175, 918, 320, 120 and 640 Pa for basement membrane, stroma, lymph, lymph node and liver, respectively) [2,4] and, while most of these cells die during their journey, few resist and can generate metastases [13]. Whether soft tissue increases malignancy or in contrast limits invasive cell spreading remains an open question. Using polyelectrolyte multilayers films (PEM) [14-18], we revealed that human SW480 colon cancer cells displayed increasing frequency in chromosomal segregation abnormalities when cultured on substrates with decreasing stiffness (Figure 1) and [3]. In the present paper, we report that substrates with stiffness of 50 kPa and lower cause massive death of mitotic cells but that few cells resist and achieve mitosis by overcoming abnormal chromosomal segregation. For this purpose, synchronized SW480 cells were seeded on a series of films made of a poly-L-lysine/hyaluronic acid (PLL/HA)_24_ stratum capped by poly(styrene) sulfonate/polyallylamine (PSS/PAH)_*n*_ strata (*n* = 0, 1 and 2; increasing *n* increases substrate stiffness [19]) and followed by live-cell imaging. 

## Results and Discussion

### 1. Influence of soft substrate on the tumor mitotic progression

**Figure 2:**
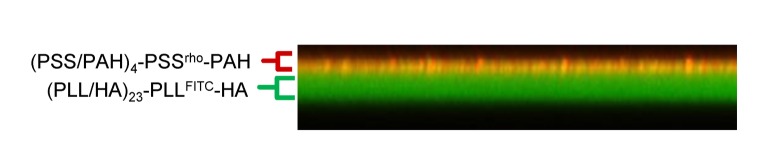
PEM characterization. Vertical section image of a (PLL/HA)_23_-PLL^FITC^-HA-(PSS/PAH)_2_-PSS^Rho^-PAH multilayered film observed by CLSM.

**Figure 3:**
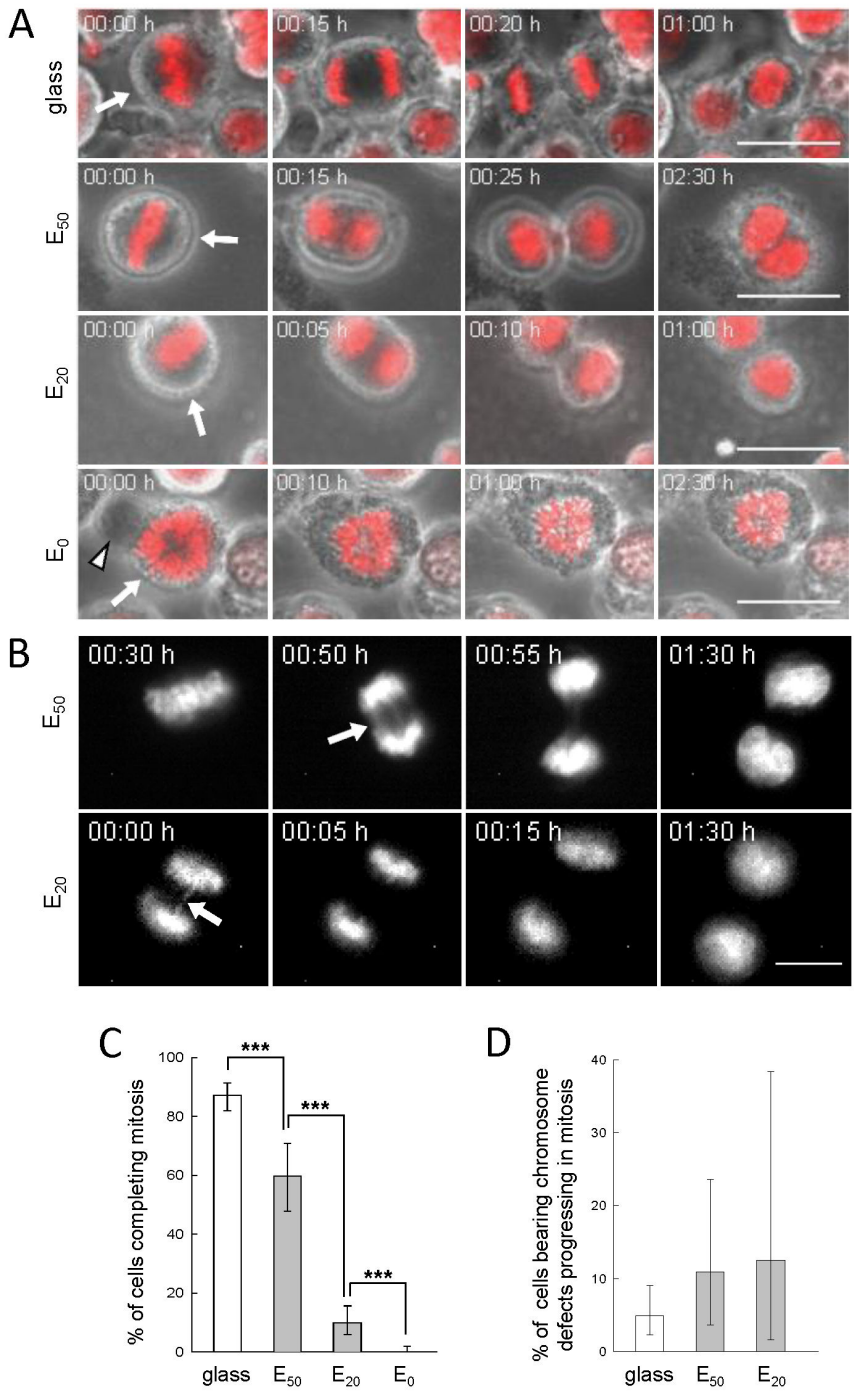
SW480 cells progression through mitosis with respect to elastic moduli of the substrate. A) After seeding of synchronized SW480 cells on glass, E_50_, E_20_ and E_0_, time-lapse image were taken every 5 min for 2h30; representative images are shown. Arrow indicates the initial position of the mitotic cell. Merged images of fluorescence (DNA in red) and phase contrast (in gray); scale bar: 20 µm. B) Time-lapse monitoring chromosome segregation in SW480 cells performed as described in A. Representative images of chromosome segregation abnormalities are displayed for E_50_ and E_20_; scale bar : 10 µm. C) Percentage of SW480 cells completing mitosis considered in A, determined on 2 pooled independent experiments. Fisher Exact Test shows that the proportion of cells completing mitosis on E_50_ (46 cells on 77 cells in mitosis) is significantly smaller from the proportion on glass (185/212, *p* < 0.001). The proportion on E_20_ (16/161) is significantly smaller than that on E_50_ (*p* < 0.001) and the proportion on E_0_ (0/146) is significantly smaller than that on E_20_ (*p* < 0.001). D) Percentage of cells with lagging chromosomes. In C, D, the error bars represent 95% confidence intervals.

**Figure 4:**
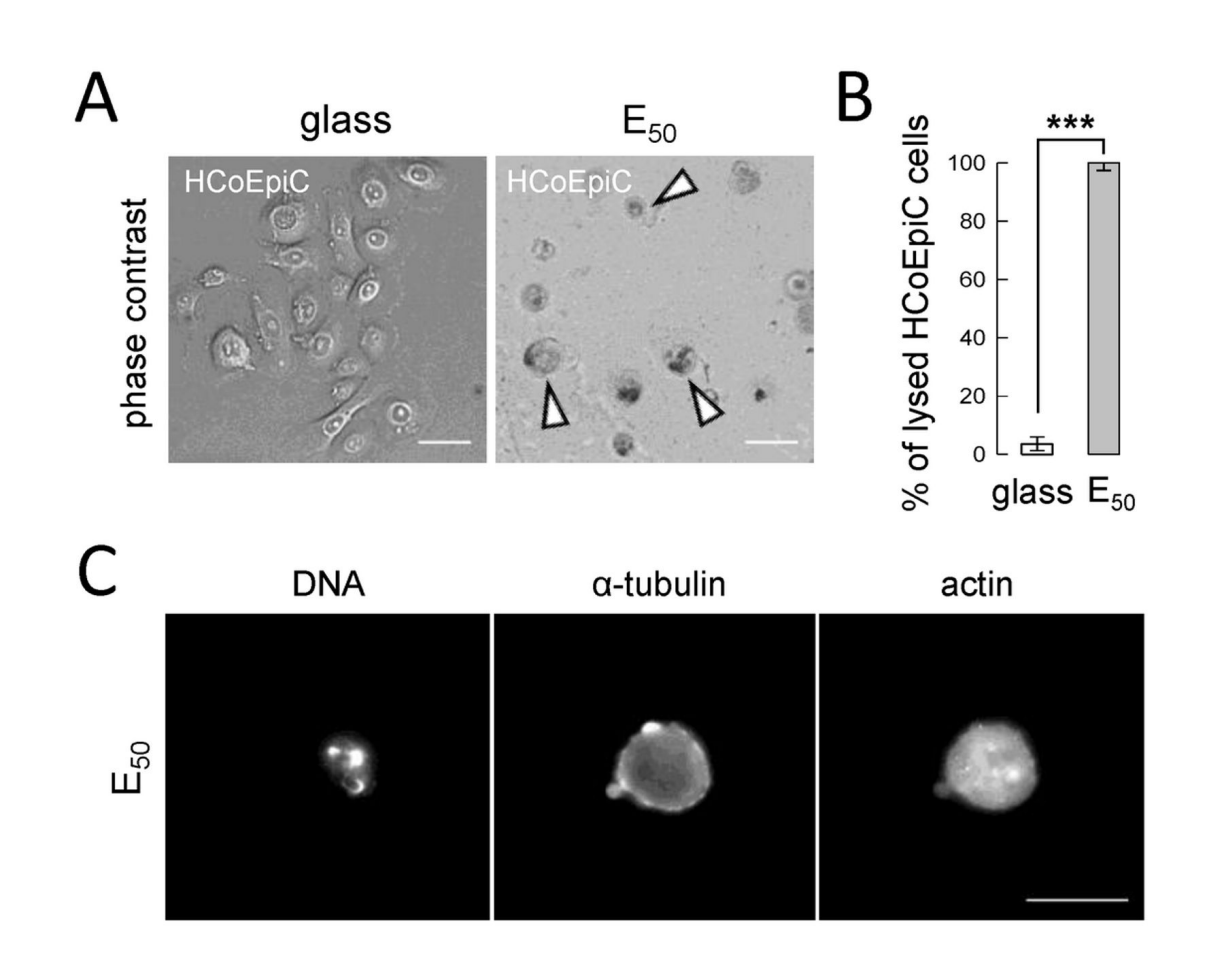
Behavior of asynchronous HCoEpiC cells on E_50_. A) Representative images of HCoEpiC cells after 6h of culture on glass and on E_50_, arrowhead indicates a lysed cell; scale bar: 50 µm. B) Percentage of lysed HCoEpiC cells considered in A, determined on 2 pooled independent experiments. Fisher Exact Test shows proportion of lysed cells on glass (5/142) is significantly smaller than that on E_50_ (112/112, *p* < 0.001). The error bars represent 95% confidence intervals. C) Fluorescence images of DNA, α-tubulin and actin after 6h of culture on E_50_ from fixed HCoEpiC cells; scale bar: 20 µm.

To determine whether tumor cells are able to progress through mitosis on very soft substrates, SW480 cells, synchronized using the mitotic shake-off method, were seeded on PEM films with decreasing stiffness (Young moduli decreasing from 50 down to 0 kPa, Table 1) and followed by live-cell imaging during 2h30. The films were composed of a (PLL/HA)_24_ stratum capped by a second (PSS/PAH)_*n*_ stratum (*n* = 0, 1 and 2). A typical example of confocal z-section observation of a film composed of PLL/HA stratum and PSS/PAH capping is displayed in Figure 2. Even though the surface chemistry of glass and of the polyelectrolyte multilayers is different, we demonstrated in a previous work that the amount of FBS proteins deposited on the surface does depend neither on the surface chemistry nor on the number of layers constituting the polyelectrolyte multilayers film [16]. Moreover, cells feel essentially the proteins from the serum that adsorb on the surface prior to cell deposition. Thus the main parameter that changes between glass, E0 and E50 / E20 is rigidity and it should be at the origin of the differences in cell behaviour observed in our system. On E_0_, cells rapidly went through a lytic process, as indicated by the release of cytoplasm in the culture medium observed in 100% of the cases (Figure 3A, arrowhead in row E_0_, Movie S1). However, on E_50_ and E_20_ substrates, the follow up of chromosome segregation, DNA decondensation and cytokinesis (Figure 3A and Movie S2-S4) revealed that respectively 60% and 10% of cells were able to achieve mitosis in 2h30 (Figure 3C). The remaining cells either lyse (18% on E_50_ and 88% on E_20_) or kept blocked in mitosis (22% on E_50_, and 2% on E_20_). Tilghman et al. showed that cancer cells cultured on soft polyacrylamide gel substrates exhibited a longer cell cycle, due to an extension of the G1 phase of the cell cycle, compared to cancer cells growing on more stiff substrates [20]. In order to demonstrate that the significant proportion of SW480 cells able to progress in mitosis on E_50_ was related to the cancerous nature of these cells, their behavior were compared to those of the non-cancerous human colonic epithelial cells HCoEpiC. Production of mitotic HCoEpiC cells by mechanical shakeoff was greatly reduced. Thus, standard asynchronous HCoEpiC cell cultures were used to investigate the influence of E_50_ on human colonic epithelial cells. The results show that after 6h of culture on E_50_, asynchronized HCoEpiC cells adopted a round shaped morphology (Figure 4A) unlike the spread shape of these cells observed on glass (Figure 4A). On E_50_, HCoEpiC cells went through a lytic process, as indicated by the release of cytoplasm in the culture medium in 100% of the cases (Figure 4A). Some of these cells showed fragmentation of their nucleus suggesting death by apoptosis (Figure 4C). Consistent with these observations, no assembly of microtubules and actin filaments could be observed by immunofluorescence experiments using antibody specific for α-tubulin and phalloidin (Figure 4C). All interphase HCoEpicC cells died on E_50_ (Figure 4B) revealing that these cells are obviously unable to progress in the cell cycle and to re-enter in mitosis. We can point out that our studies were performed on substrates with Young moduli in the range of 1-50 kPa and we observed that non-cancerous cells could not survive on such soft substrates. This result seems at first sight contradictory with the observation of cell division in colon whose Young modulus is 2 - 25 kPa [21]. Yet, in colon cells are in a specialized 3D tissue with cell/cell contacts which strongly influence cell behaviour. Such an effect is not reproduced in our 2D in vitro experiments. Nevertheless, our in vitro model is more appropriate for single tumour cells or small groups of cells that escape the tumour mass to invade the stroma and engage in the process leading to metastasis formation after a long journey through tissues of very different Young moduli. Altogether, our results show that the decrease of the stiffness correlates with an increase in cell lethality in vitro for healthy as well as cancerous cells. Nonetheless, very importantly, we observed that some cancer cells can escape from very soft substrates and can achieve mitosis. These findings are highly relevant for both normal homeostasis of solid tissues and for pathological settings.

**Table 1:** Apparent elastic modulus of (PLL/HA)_24_-(PSS/PAH)_*n*_ with *n* = 1 and 2.

**Architecture**	**E_ap_ (kPa)^a^**	**Notation**
(PLL/HA)_24_	~0	E_0_
(PLL/HA)_24_ – (PSS/PAH)_1_	~20	E_20_
(PLL/HA)_24_ – (PSS/PAH)_2_	~50	E_50_

The films were composed of a hyaluronic acid/poly-L-lysine (HA/PLL)_24_ stratum capped by a second poly(styrene) sulfonate/polyallylamine hydrochloride (PSS/PAH)_*n*_ stratum. The films were characterized by their Young’s modulus (*E*_ap_: apparent elastic modulus), determined by atomic force microscopy nano-indentation experiments, which would correspond to the real elastic modulus of the layer if it behaved strictly elastically. The elastic modulus of the native (PLL/HA)_24_ architecture is about 0 kPa. ^a^ Values taken from [19].

### 2. Behavior of tumor cells bearing chromosome segregation abnormalities

Time-lapse monitoring chromosome segregation showed that SW480 cells seeded on E_50_ and on E_20_ displayed lagging chromosomes (Figure 3B, arrows) indicating defects in chromosomal segregation mechanisms. In late telophase, the nuclei reformed and cytokinesis completed (Figure 3B, 3D and Movie S5 and S6). Among the SW480 cells progressing through mitosis 4.8%, 11% and 13% showed chromosome segregation abnormalities on glass, E_50_ and E_20_, respectively. These results showed that despite increasing frequency of abnormalities induced by soft substrates, some SW480 cancer cells are able to progress through mitosis. Which is consistent with observation made on rigid substrates that cells having deficient spindle checkpoint mechanisms may proceed through mitosis even in the presence of chromosomes misconnected to the spindle [22]. 

### 3. The β1-integrin engagement by mitotic tumor cells in response to soft substrates

**Figure 5:**
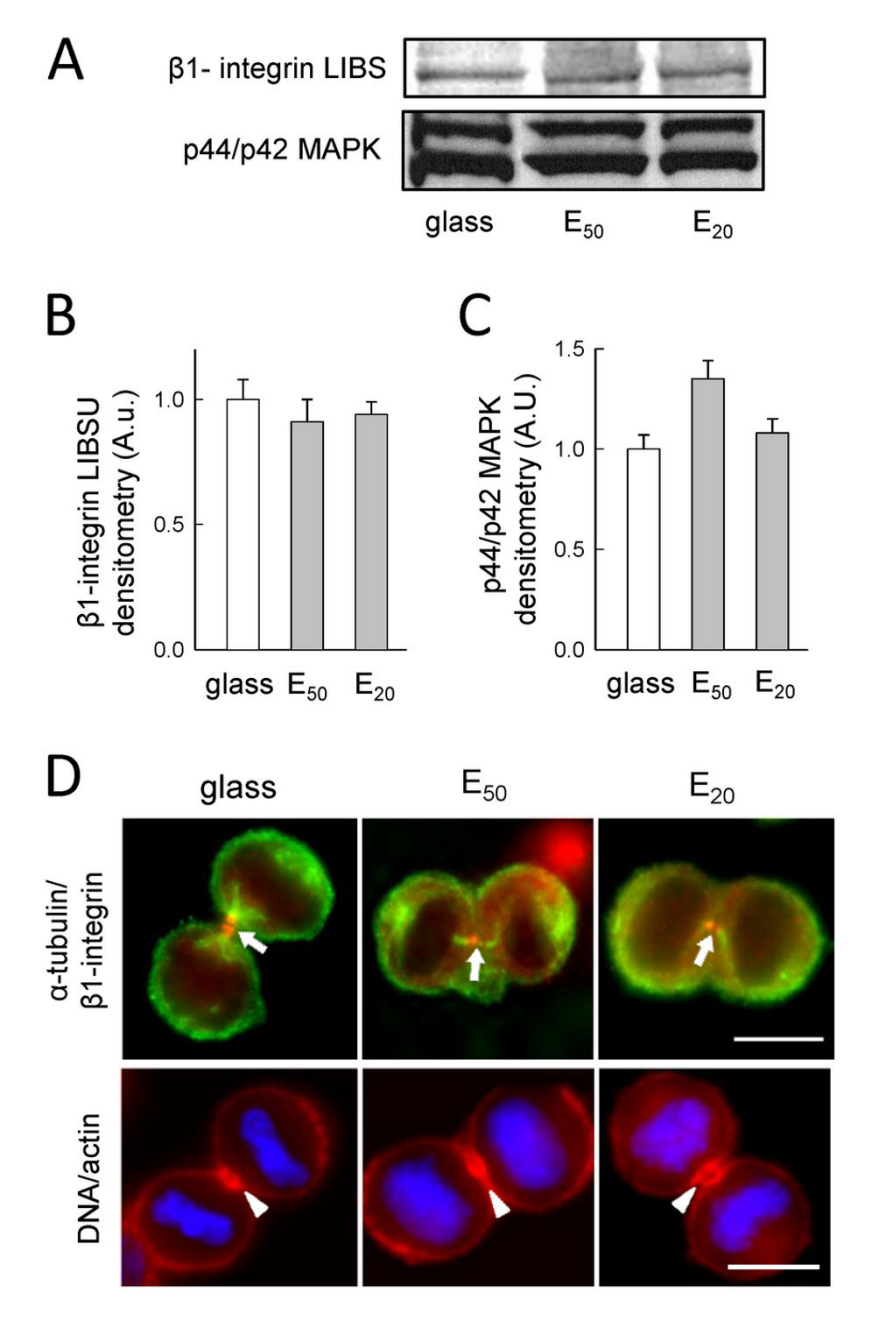
β1-integrin engagement of mitotic SW480 cells with respect to soft substrates. A) Western blots of β1-integrin LIBS and MAPK protein for SW480 cells seeded 30 min post-synchronization on glass, E_50_ and E_20_. Histogram shows the corresponding scans from B) β1-integrin LIBS and C) p44/p42 MAPK control loading. Representive results from 2 independent experiments (the error bars represent the s.e.m.; an arbitrary value of 1 was attributed to the mean value corresponding to cells on glass). D) α-tubulin and β1-integrin distribution 30 min post-synchronization on glass, E_50_ and E_20_ from fixed SW480 cells, superposition of cells with anti-α-tubulin (green) and anti-β1-integrin (red) (α-tubulin/ β1-integrin). Arrow indicates a fine point of β1-integrin concentrated on the mid-body. Representative images are shown for 2 independent experiments for a total of 10 cells for each condition; scale bar : 10 µm. On glass, E_50_ and E_20_ from fixed SW480 cells, superposition of cells with DNA (blue) and actin (red) (DNA/actin). Arrowhead indicates actin accumulation in the cell mid-zone. Representative images are shown for 2 independent experiments for a total of 10 cells for each condition; scale bar: 10 µm.

Interactions with the extracellular matrix through integrins have been shown to influence different aspects of mitotic spindle organization [23,24]. In particular, it is well known that mitotic cells become rounded in preparation for cytokinesis and remain attached to the substrate through retraction fibres via integrin engagement [25]. The retraction fibres provide resistive force that propagates within microtubules to orient the mitotic spindle [23-26]. We thus investigated the effects the different substrate stiffness on β1-integrin in SW480 cells. In response to soft substrates (E_50_ and E_20_), β1-integrin engagement measured by ligand-induced binding sites (activated β1-integrin LIBS) using conformational-specific antibodies was unchanged compared to glass substrate (Figure 5A-C, the MAPK lane correspond to control loading). Interestingly, these data are in contrast with a previous study using non-tumor PtK2 cells showing progressively decreases of β1-integrin engagement on soft substrates [3]. These observations are in accordance with other studies demonstrating that murine breast cancer cells retain high levels of active β1-integrin after 24h of culture on a soft substrate [27]. Integrin accumulation in the cell mid-zone is also necessary to induce mitotic cell adhesion and to support cytokinesis by providing mechanical anchoring for the contractile actomyosin ring [28]. To examine the localization of β1-integrin, immunofluorescence performed in cells in telophase revealed β1-integrin accumulation in the mid-body, on E_50_ and on E_20_, like on glass (Figure 5D). On glass, E_50_ and E_20_ actin accumulated also in this cell mid-zone (Figure 5D). These results suggest continuous β1-integrin engagement during mitosis despite the soft substrates, compatible with the involvement to support cytokinesis.

### 4. Mitotic spindle organization of tumor cells in response to soft substrates

**Figure 6:**
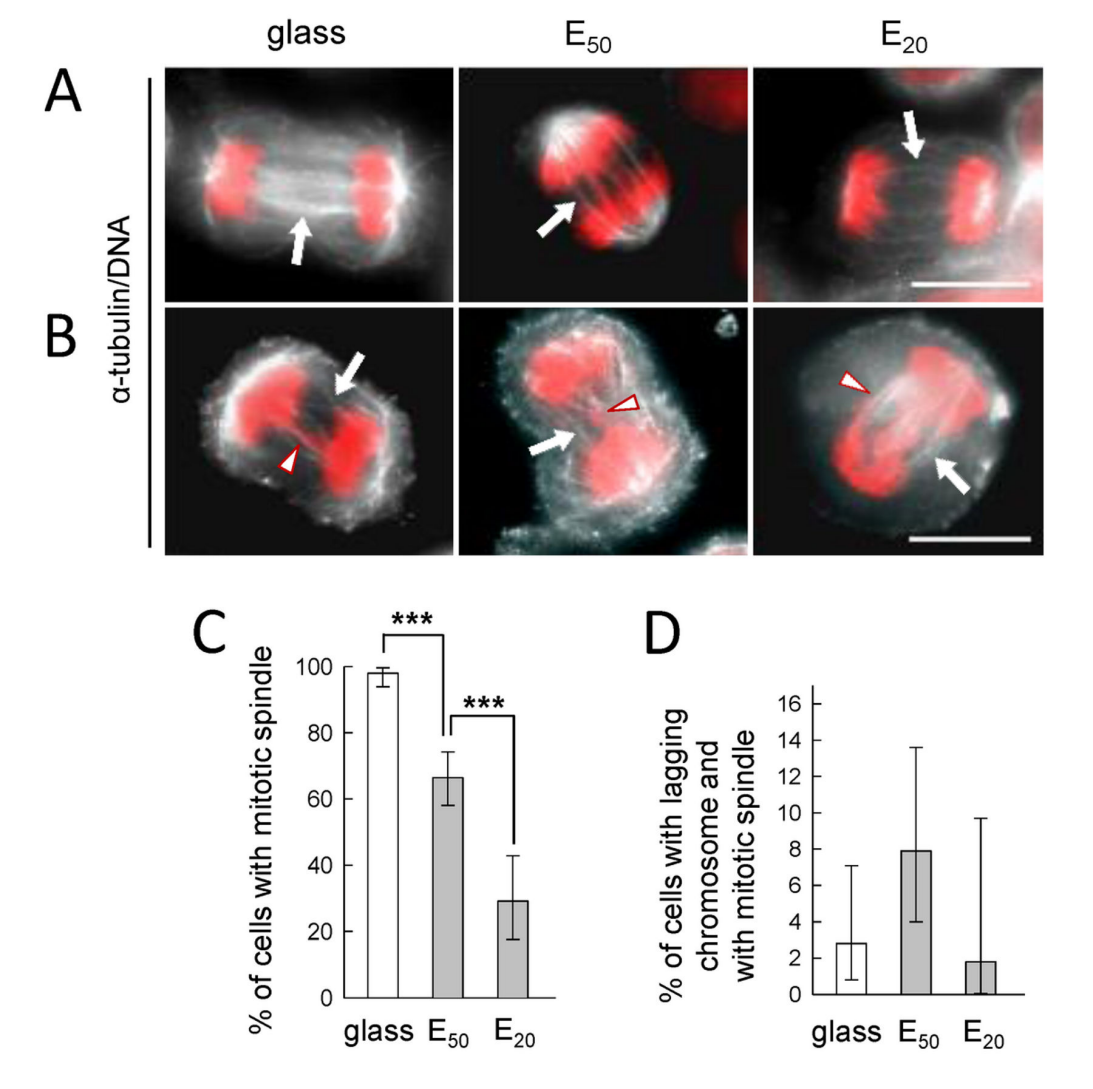
Mitotic spindle organization with respect to soft substrates. SW480 cells 30 min-2h post-synchronization on glass, E_50_ and E_20_ from fixed cells. Images are superimposed with anti-α-tubulin (white) and Hoechst for DNA (red) (α-tubulin/DNA). Arrow indicates the mitotic spindle, without anomaly (A), with anomaly (B), arrowhead indicates the anomaly; scale bar : 10 µm. C) Percentage of SW480 cells with mitotic spindle from A, determined on 3 pooled independent experiments. Fisher Exact Test shows that the proportion of cells with a mitotic spindle on E_50_ (93/140) is significantly different from the proportion on glass (139/142, *p* < 0.001). The proportion on E_20_ (16/50) is significantly smaller than that on E_50_ (93/140, *p* < 0.001). D) Percentage of cells bearing chromosome segregation abnormalities, among the cells with mitotic spindle considered in C. In C and D, the error bars represent 95% confidence intervals.

 We next checked mitotic spindle assembly on soft matrices by immunofluorescence with anti-α-tubulin and DNA staining with Hoechst. The mitotic spindles of SW480 cells, visible on stiff substrates (glass), were preserved on E_50_ and even on E_20_ (Figure 6A and B). This result contrasts with that observed for non-cancerous mitotic PtK2 cells seeded on soft matrix since for Young modulus ≤ 50 kPa, the mitotic spindle could not be observed [3]. Consistent with our living cell analysis (Figure 3B and D), abnormal chromosome segregation events could be observed (Figure 6B and D), however, without evidence for multipolar or monopolar spindles on the different substrates probably due to β1-integrin engagement maintained on the very soft substrate. Indeed, multipolar spindles were observed for cells bearing mutated β1-integrin [23]. 

### 5. Rac1 expression of tumor cells in response to soft substrates

**Figure 7:**
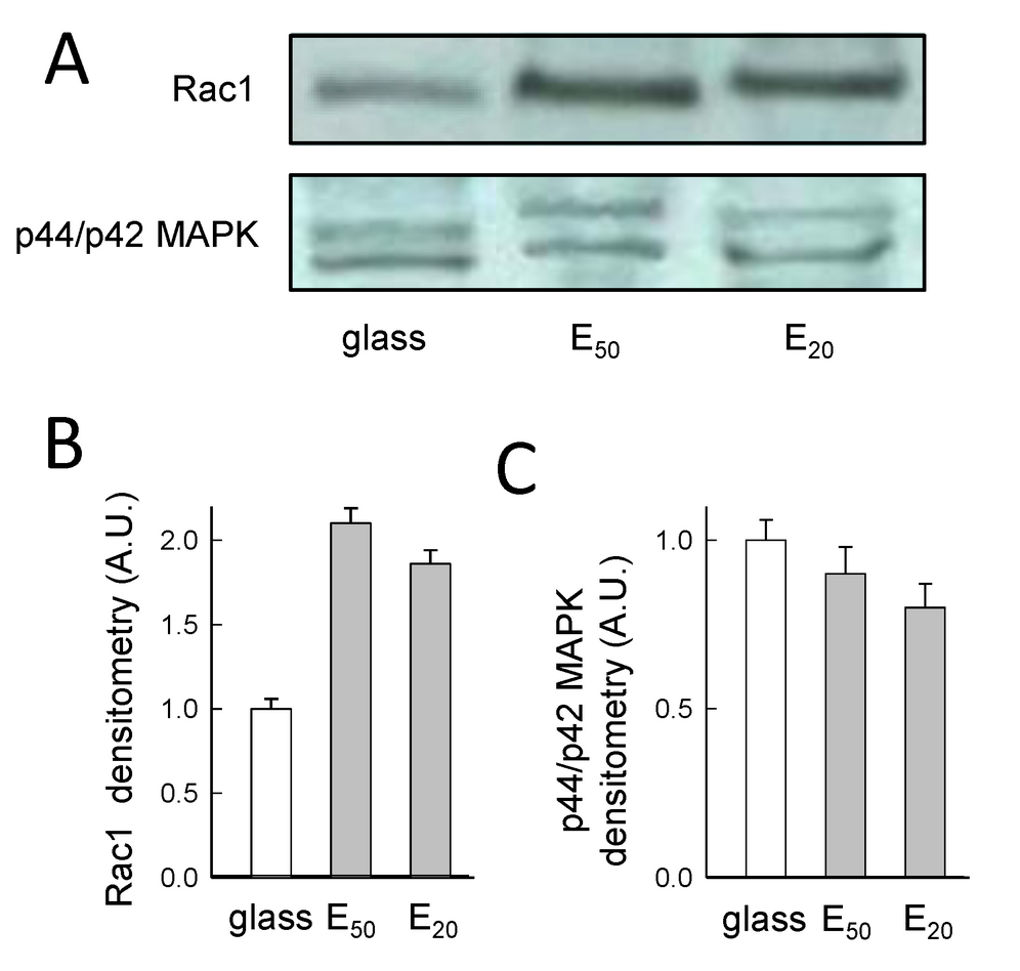
Rac1 expression of mitotic SW480 cells with respect to soft substrates. A) Western blots of Rac1 and MAPK protein for SW480 cells on glass, E_50_ and E_20_, 30 min post-synchronization. Histogram shows the corresponding scans for Rac1 (B) and for p44/p42 MAPK (C). Representive results from 2 independent experiments (the error bars represent the s.e.m.; an arbitrary value of 1 was attributed to the mean corresponding to cells on glass).

Rac1, proteins of the small GTPase Ras family, are involved in the orientation of the mitotic spindle and its activity increases at the plasma membrane of polar sides during cytokinesis [29]. We further synchronized a collection of SW480 cells and analyzed Rac1 expression through Western blot experiments [29]. There were no differences in Rac1 expression for mitotic SW480 cells seeded either on soft substrates (E_50_ and E_20_) or on stiff substrate (glass) (Figure 7A-C). Our results showed that mitotic tumor SW480 cells maintain Rac1 expression on soft substrates. On the contrary, we previously observed that non-cancerous PtK2 cells progressively decreases this expression on soft substrates [3]. Importantly, β1-integrin engagement and Rac1 expression maintained on soft substrates (E_50_, E_20_) were not sufficient to allow massive division of tumor SW480 cells. Indeed, on these substrates only 60% (E_50_) and 10% (E_20_) of cells are able to progress in mitosis

### 6. DNA replication activity of tumor cells in response to soft substrates

**Figure 8:**
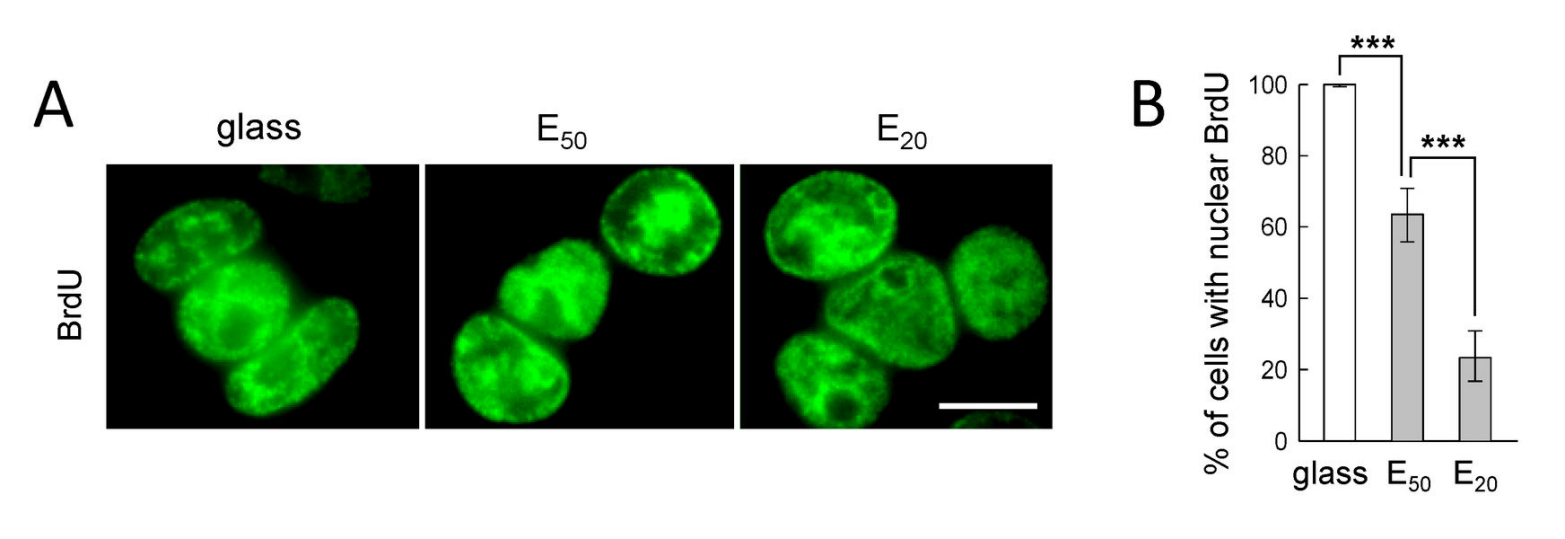
DNA Replication with respect to soft substrates. A) BrdU visualized by indirect immunofluorescence of SW480 cells 4h post-synchronization on glass, E_50_ and E_20_; scale bar: 10 µm. B) Percentage of cells with nuclear BrdU determined on 3 pooled independent experiments. Fisher Exact Test shows that the proportion of cells with nuclear BrdU E_50_ (108/170) is significantly smaller than that on glass (400/400, *p* < 0.001) and the proportion on E_20_ (35/150) is significantly smaller than that on E_50_ (*p* < 0.001). The error bars represent 95% confidence intervals.

To determine whether SW480 cells that were able to progress through mitosis were capable of reenter cell cycle, we investigated their capability to undergo DNA replication 4h after being seeded on different substrates. SW480 cells seeded on E_50_ and E_20_ show site of replication uniformly distributed in the nucleus (Figure 8A) with respectively 60% and 23% of cells with nuclear BrdU signal (Figure 8B). It is noteworthy that the percentages of SW480 cells incorporating BrdU correlated with the percentage of cells achieving mitosis on E_50_ and E_20_ (Figures 3C and 8B), suggesting that SW480 cells able to complete chromosome segregation on soft substrates are further able to undergo a new cycle of DNA replication. 

## Conclusion

In conclusion, we report that despite massive cell death on extremely soft substrates (E_0_), tumor cells like the SW480 colon cancer cells, even when bearing abnormal chromosome segregation, resist to the very soft substrates (E_20_ and E_50_) and achieve mitosis. These findings might be highly relevant for the pathophysiology of cancer and the dissemination of colon tumor cells. Indeed, their cancerous nature at least some of the tumor cells might help them to overcome chromosomal segregation abnormalities linked to the change in substrate stiffness and therefore escape the soft substrates to pursue their journey up to the site of metastasis formation. Moreover, this ability to overcome segregation abnormalities could result in more chromosomal rearrangements, which may contribute to increasing tumor cell aggressiveness. Further investigating the response of tumor cells to physical environmental changes may help to identify new potential targets for anticancer therapy. 

## Materials and Methods

### 1. Materials and fabrication of PEM

PLL (MW = 5.7 x 10^4^ Da, Sigma, St. Quentin Fallavier, France) and HA (MW = 4.0 x 10^5^ Da, BioIberica, Barcelona) were used for buildup (PLL/HA)_24_ films, and PSS (MW = 7.0 x 10^4^ Da, Sigma, St. Quentin Fallavier) and PAH (MW = 7.0 x 10^4^ Da, Sigma) for (PSS/PAH)_*n*_ capping films (*n* corresponds to the number of layer pairs), which were deposited on top of (PLL/HA)_24_ strata. PLL, HA, PSS, and PAH were dissolved at 1 mg/mL in a buffer solution containing 150 mM NaCl and 20 mM of tris(hydroxymethyl)-aminomethan (TRIS, Merck) at pH 7.4, and all rinsing steps were performed in the same buffer. (PLL/HA)_24_ strata and (PSS/PAH)_*n*_ capping films were prepared using a dipping machine (Dipping Robot DR3, Riegler & Kirstein GmbH, Berlin, Germany), on glass slides (VWR Scientific, Fontenay sous Bois, France). The rigidity of the (PLL/HA)_24_-(PSS/PAH)_*n*_ film increases with the number of deposited PSS/PAH layer pairs (Table 1) [19]. The short hand notations E_0_, E_20_ and E_50_ are for (PLL/HA)_24_, (PLL/HA)_24_-(PSS/PAH)_*n*_ films with *n* = 0, 1 and 2, respectively. 

### 2. PEM characterization

CLSM observations were performed with Zeiss LSM 510 microscope using x40/1.4 oil immersion objectif. FITC-fluorescence detected after excitation at 488 nm with cutoff dichroic mirror 488 nm and emission band-pass filter 505-530 nm. Rho-fluorescence detected after excitation at 543 nm, dichroic mirror 543 nm, and emission long pass filter 585 nm. 

### 3. Cells and synchronization

Colorectal adenocarcinoma epithelial SW480 cells (ATCC, CCL-228) were grown in RPMI-1640 medium (Invitrogen) supplemented with glutamax, 10% FBS (Invitrogen), 100 µg/mL penicillin, 100 µg/mL streptomycin (Invitrogen), 0.025 U/mL insulin, 50 mg/mL hydrocortisone and 1.25 mg/mL G418 maintained at 37°C with 5% CO_2_. Three days prior to synchronization, cells were replated at 1.2x10^4^ per cm^2^. Cells were synchronized by mechanical shakeoff. Mitotic cells were centrifuged (800 *g*, 7 min), resuspended in culture medium, and replated at 1.2x10^4^ per cm^2^ on film-coated coverslips for further analyses. Human Colonic Epithelial Cells (HCoEpiC, ScienCell Research Laboratories) were grown on Colonic Epithelial Cell Medium (CoEpiCM, ScienCell Reserach Laboratories) supplemented with colonic epithelial cell growth supplement (CoEpiCGS, ScienCell Research Laboratories) and with penicillin/streptomycin solution (P/S, ScienCell, Research Laboratories) maintained at 37°C with 5% CO_2_. 2 population doublings were plated at 5x10^5^ per cm^2^ on substrates for further analyses.

### 4. Immunolabeling

Cells were fixed/permeabilized in 3.7% (w/v) PFA in PBS plus 0.1% Triton X-100 for 15 min and blocked with 10% decomplemented FBS (Invitrogen). Cells were incubated with β1-integrin (dilution 1:20, Santa Cruz followed by rhodamin-conjugated secondary antibody (dilution 1:250, Santa Cruz), or with anti-α-tubulin (dilution 1:100, Santa Cruz) followed by FITC-conjugated secondary antibody (dilution 1:500, AnaSpec, CA) and DNA was revealed with Hoechst 33258 (20 µg/mL, Sigma). For DNA replication studies, cells previously grown with BrdU (37°C) (1:50, RPN 201, GE Healthcare) were fixed/permeabilized, incubated with anti-BrdU and DNase for 1 h at 37°C (dilution 1:100, RNP 202, GE Healthcare), followed by TRITC-conjugated secondary antibody (1:500, AnaSpec). 

### 5. DNA Replication

1.2×10^4^ synchronized cells were seeded per cm^2^ and incubated with BrdU (37°C) (1:50; RPN 201, GE Healthcare). Cells were fixed/permeabilized in 3.7% PFA in PBS plus 0.5% Triton X-100 for 15 min. After washing with PBS, cells were incubated with anti-BrdU and DNase for 1 h at 37°C (diluted 1:100; RNP 202, GE Healthcare). After washings with PBS, cells were incubated with TRITC-conjugated secondary antibody (1:500; AnaSpec). 

### 6. Fluorescence microscopy

Samples were mounted in VectaShield (Vector Laboratories, Burlingame, CA). fluorescence images were captured using Nikon Elipse TE200 with 63× PL APO (1.4 NA) objectif equipped with Nikon Digital Camera (DS-Q11MC with NIS-Elements softwares), and processed with ImageJ (http://rsb.info.nih.gov/ij/).

### 7. Live-cell imaging

For dividing assays, cells were incubated 20 min with Hoechst 33242 (0.1 µg/mL, Sigma) prior to mechanical shakeoff, replated at 1.2x10^4^ per cm^2^ on film-coated coverslips and mounted in a Ludin Chamber (Life Imaging Services, Basel Switzerland) at 37°C, 5% CO_2_, on a Leica DMIRE2 microscope equipped with a 40× HCX PL APO PH2 (0.75 NA) objective and a Leica DC350FX CCD (Leica FW4000 software). Images were acquired every 5 min for 2h30, by fluorescence and phase contrast.

### 8. Western Blot

Cells were seeded on surfaces (Nunc) at 2×10^5^ per cm^2^ and incubated for 30 min post-synchronization in culture medium at 37°C. Cells were lysed in 20 mM Tris-base, pH 8, (0.15 M NaCl, 2 mM EDTA, 1 % NP-40, 10% glycerol, 1 mM sodium orthovanadate containing 1% of protease inhibitor cocktail; Sigma). Extraction mixtures are rocked at 4°C and centrifuged (3 min, 13k rpm at 4°C). Protein concentration was determined using DC protein assay (Bio Rad, USA). Equal amounts of total protein extracts were subjected to SDS PAGE (NuPAGE, Invitrogen, France) and transferred onto nitrocellulose membranes (Iblot Transfer Stack, Invitrogen, USA) blocked in T- TBS (0.1% Tween 20, 50 mM Tris-base, pH 7.6, 0.15 M NaCl) containing 1% BSA (Euromedex, France) and probed overnight at 4°C. Blots were incubated 2h with primary antibody: β-integrin activated LIBS, (clone B44) (diluted 1:1000, Millipore), anti-Rac1 (diluted 1:1000, Millipore) and p44/p42 MAPK (diluted at 1:1000, Cell Signaling, USA) were used. Blots were incubated for 2 h with HRP-conjugated anti-rabbit, anti-mouse antibodies (diluted 1:2000; GE Healthcare). Bands were detected using ECL Western Blotting Analysing System kit (RNP2109, GE Healthcare). Autoradiographs were quantified with Kodak Digital Science 10 Software. Representative mean values of at least two independent experiments with standard errors (four time-points per band) are presented.

## Supporting Information

Movie S1 **Movie corresponding to Figure 3A for SW480 cells on E_0_.**
(AVI)

Movie S2 **Movie corresponding to Figure 3A for SW480 cells on E_50_.**
(AVI)

Movie S3 **Movie corresponding to Figure 3A for SW480 cells on E_20_.**
(AVI)

Movie S4 **Movie corresponding to Figure 3A for SW480 cells on glass.**
(AVI)

Movie S5 **Movie corresponding to Figure 3B for SW480 cells on E_50_.**
(AVI)

Movie S6 **Movie corresponding to Figure 3B for SW480 cells on E_20_.**
(AVI)
